# Analyzing Possible Shifts in the Climatic Niche of *Pomacea canaliculata* Between Native and Chinese Ranges

**DOI:** 10.3390/biology14091127

**Published:** 2025-08-25

**Authors:** Ran Zhang, Yue Gao, Rui Wang, Shigang Liu, Qianqian Yang, Yuan Li, Longshan Lin

**Affiliations:** 1 Third Institute of Oceanography, Ministry of National Resource, Xiamen 361005, China; iamzhangran@163.com (R.Z.);; 2 Key Laboratory of Evolution and Marine Biodiversity (Ministry of Education), Institute of Evolution and Marine Biodiversity, Ocean University of China, Qingdao 266003, China; 3 Zhejiang Provincial Key Laboratory of Biometrology and Inspection & Quarantine, College of Life Sciences, China Jiliang University, Hangzhou 310018, China

**Keywords:** *Pomacea canaliculata*, invasion ecology, niche dynamics, invasive aquatic species, climate change, invasion behavior

## Abstract

The impact of invasive alien species (IAS) is one of the direct factors causing global biodiversity decline and economic losses, and predicting the potential invasion risks of invasive species is crucial for developing prevention and control strategies. In recent years, an increasing number of studies have shown that invasive species undergo rapid shifts in climate niche in invaded areas. Accurately quantifying the dynamic shifts in the climate niche of invasive species in invaded areas is crucial for developing a more accurate framework for early warning of invasive species risks. *Pomacea canaliculata* is a freshwater snail found in South America and has become one of the most aggressive aquatic species in the world. Since its introduction to China in 1981, it has rapidly spread and caused multiple serious damages to agriculture, ecology, and public health. Therefore, based on multi-source distribution data of *P. canaliculata*, this study calculated the climate niche overlap by Schoener’ s *D*, quantified the niche shifts between the *P. canaliculata* in native and invaded areas (China) via the COUE scheme (a unified terminology representing niche centroid shift, overlap, unfilling, and expansion), and analyzed their changes on a time scale. The results revealed that there have been significant climate niche shifts (Schoener’s *D* < 0.2, niche similarity tests *p* > 0.01, niche equivalence tests *p* < 0.01) between the native and invaded areas (China) of *P. canaliculata*, which does not support the climate niche conservation hypothesis. The minimum temperature of the coldest month (Bio 6) and precipitation seasonality (Bio 15) were the key climate variables driving the climatic niche shift, and *P. canaliculata* can survive in colder and more arid regions than their native counterparts. The changes in the niche shifts in *P. canaliculata* on a time scale show significant temporal heterogeneity, and its invasion behavior in China presents a discontinuous and phased expansion pattern, with strong adaptability to new environments. The results are of great significance for the future development of more accurate ecological niche model (ENM), the formulation of more targeted prevention and control strategies, and the study of adaptive evolution mechanisms of invasive species.

## Simple Summary

This study investigates the climatic niche shifts in the invasive species *Pomacea canaliculata* between its native and invaded area (China), and quantitative analysis was conducted. The aim is to explore whether the climate niche has changed after *Pomacea canaliculata* invasion in China, and analyze its changes in the time series. The results revealed that there has been a significant climate niche shifts between the native and invaded area (China) of *Pomacea canaliculata*, which does not support the climate niche conservation hypothesis. *Pomacea canaliculata* can survive in colder and drier regions than their native counterparts, demonstrate strong environmental adaptability. The findings are crucial for enhancing invasive species risk assessment models, developing effective control strategies, and exploring the adaptive evolution mechanisms of invasive species.

## 1. Introduction

Owing to the increase in human activities, thousands of species have been introduced into areas beyond their native range, leading to a sharp decline in biodiversity, and affecting social health and the economy [1,2]. Invasive species have a widespread negative impacts on ecosystems and biological populations, affecting ecosystem services and reducing the abundance of native species through mechanisms such as predation, hybridization, and competition [3]. Once they form a viable population, eradicating them is often difficult [4,5], and it is often impossible to detect them in a timely manner when the species first invades [3]. Freshwater accounts for only 0.01% of the world’s water resources, but its biodiversity per unit surface area is greater than that of terrestrial and marine ecosystems [6]. Among them, freshwater invasive mollusks constitute one of the most dangerous groups [7,8]. *Pomacea canaliculata* is a freshwater snail native to the Rio de la Plata Basin in South America. This species is in the family Ampullaridae and is a highly invasive species of freshwater snail [9]. There are three ways in which *P. canaliculate* is harmful. First, it is the main pest of aquatic crops such as rice, which can cause crop yield reduction by gnawing on seedlings, resulting in considerable economic losses [10,11]. Second, the *P. canaliculata* can damage the structure and function of wetland ecosystems, threatening the survival of local species by altering nutrient cycling and primary productivity [12,13]. In addition, *P. canaliculata* is also an intermediate host of *Angiostrongylus cantonensis*, which may cause human eosinophilic meningitis and pose a serious threat to public health [14,15]. Therefore, *P. canaliculata* has become one of the most invasive aquatic organisms worldwide [16,17,18], and has been listed as one of the 100 most serious invaders by the International Union for Conservation of Nature (IUCN) [17].

At present, research on the invasion of *P. canaliculata* has focused mainly on the following aspects: first, the invasion ecology of *P. canaliculata*, including diffusion pathways, invasion mechanisms, and their impact on local ecosystems [16,19]; second, its physiological adaptability, such as tolerance to environmental stresses such as low temperature and drought [15,20]; and third, the ecological niche model (ENM) has been used to predict its potential distribution range and assess its future invasion risk [21,22]. The use of the ENM to evaluate the future distribution of *P. canaliculata* can help us develop management strategies for *P. canaliculata* invasion. Because the ecological niche describes the range of environmental conditions in which a specific species appears, it reveals the relationship among the distribution, environmental variables, and potential range shifts in invasive alien species in the context of global climate change, providing an important foundation for invasive ecology [23,24]. However, one of the key underlying assumptions of ENMs is the climatic niche conservatism hypothesis [25,26]. However, not all invasive species exhibit niche conservatism during geographical expansions, and a shift in the niche can be the result of adaptive changes, the release of natural enemies, the disappearance of dispersal constraints, or tolerance to conditions presently unavailable in the native range [27,28]. If the target species does not follow the assumption of the niche conservation, predictions based on the ENM may be misleading, underestimating the potential distribution of invaded areas and affecting the effective management of species invasion. Climatic niche shifts may mean that these species adapt to new climates during the invasion process, and invasive species with stronger niche shifts may have greater invasion risk or potential. Therefore, the climatic niche shifts in invasive species is also an important aspect of invasive ecology research [29,30,31].

Most previous studies have assumed that the climatic niche of the *P. canaliculata* is conserved between its native and invaded areas. However, recent studies have shown that *P. canaliculata* may exhibit significant niche shifts during invasion and can adapt to a wider range of climatic conditions [22,32]. *P. canaliculata* was introduced to China in the 1980s and was initially raised for economic reasons for food. Owing to their poor meat texture and limited market demand, *P. canaliculata* has been abandoned in large numbers and have quickly spread in the wild [21]. At present, the distribution range of *P. canaliculata* in China is constantly expanding, gradually spreading to many places such as Guangxi, Fujian, Zhejiang, Yunnan, Shanghai, Jiangsu, Hunan and Sichuan, causing considerable economic losses [33]. However, there is currently little research on the niche shift in *P. canaliculata* after its invasion in China, which limits a comprehensive understanding of its potential for invasion in China and affects the formulation of prevention and control strategies. If a niche shift is ignored and its distribution is predicted on the basis solely of environmental data from the native area, its actual invasion range may be underestimated. In addition, against the background of global climate change, the distribution of *P. canaliculata* may further expand to high-latitude regions, and existing models have different predictions. Therefore, on the basis of the distribution data and bioclimatic data of *P. canaliculata* in its native and invaded areas (China), this study used the COUE scheme (a unified terminology representing niche centroid shift, overlap, unfilling, and expansion) [27] to analyze the shifts in the climatic niche of *P. canaliculata* in their native and invaded areas (China), as well as the main driving climate variables, and further analyzed their changes on a time scale. These research results can provide a reference for more accurate assessment of the invasion risk of *P. canaliculata* and the development of more scientific and effective prevention and control measures.

## 2. Materials and Methods

### 2.1. Occurrence Records (P. canaliculata Distribution Data)

The distribution data of *P. canaliculata* were obtained from scientific references [18,34,35,36,37,38,39,40,41,42,43,44,45,46], the Global Biodiversity Information Facility (GBIF, http://www.gbif.org/, accessed on 2 August 2025; links to all GBIF datasets can be found in Appendix A) and field collection data from scientific surveys. First, we conducted a preliminary cleaning of the collected distribution data. For the distribution data of *P. canaliculata* collected from the scientific literature and scientific survey, we verified the coordinate information using Google Earth to remove duplicate and inaccurate distribution point data; for the data obtained from the GBIF, records with latitudes and longitudes equal to zero, coordinates falling into the ocean, or near animal breeding facilities were deleted. Second, to avoid spatial autocorrelation, Moran’s index was used to exclude points less than 10 km apart, and the R package “sp. Thin” (version: 0.2.0) was used to refine all the spatial distribution data [47]. After spatial refinement, a total of 1220 geographic distribution data points were retained. It is important to clarify that the distribution data of *P. canaliculata* collected in this study predominantly originate from its distribution patterns in natural water bodies. Specifically, the survey collected data were primarily gathered from locations such as riverbanks, areas beneath trees, roadside zones, and wetland parks within urban and rural settings. The data sourced from references mainly consist of investigation findings concerning the distribution of *P. canaliculata* in natural environments. Additionally, the data downloaded from the GBIF underwent thorough processing, during which potentially erroneous distribution records were removed. Although it is not entirely possible to exclude the possibility that some of the distribution data may have been derived from artificial reservoirs or experimental rice paddies, such instances are found to be relatively scarce when compared to the data from natural water bodies. This data composition is beneficial for investigation into the climatic niche shift in *P. canaliculata* and reduces potential biases from human-induced environmental modifications.

**Figure 1:**
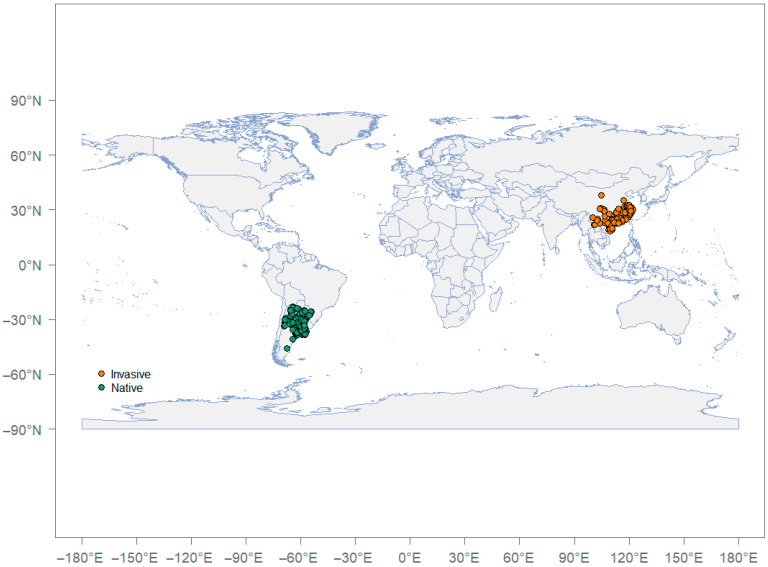
Global records of *P. canaliculata* showing the “native” and “invasive (China)” distribution utilized for niche analyses.

According to the regional scope, we divided the distribution data of *P. canaliculata* into two groups: “native distribution” and “invasive distribution (in China)”. To analyze the climatic niche shifts in the *P. canaliculata* the invaded area (China) over time, we divided the distribution data of the “invasion (in China)” group into three groups on the basis of a 10-year time period: 2000s, 2010s, and 2020s. The above process was repeated three times, and similar results were obtained each time (Figure 1). Therefore, the sample size may not significantly alter our analysis results.

### 2.2. Climatic Variables

The bioclimatic variables used in the models were obtained from the WorldClim database Version 2.1 (https://www.worldclim.org/data/worldclim21.html, accessed on 2 August 2025) at a spatial resolution of 30 s (~1 km^2^). The 19 climate variables are shown in Table 1.

To avoid including highly correlated variables in the models, multicollinearity was evaluated using Pearson pairwise correlation by means of the “Performance Analytics” statistical package in R 4.3.1.

**Figure 2:**
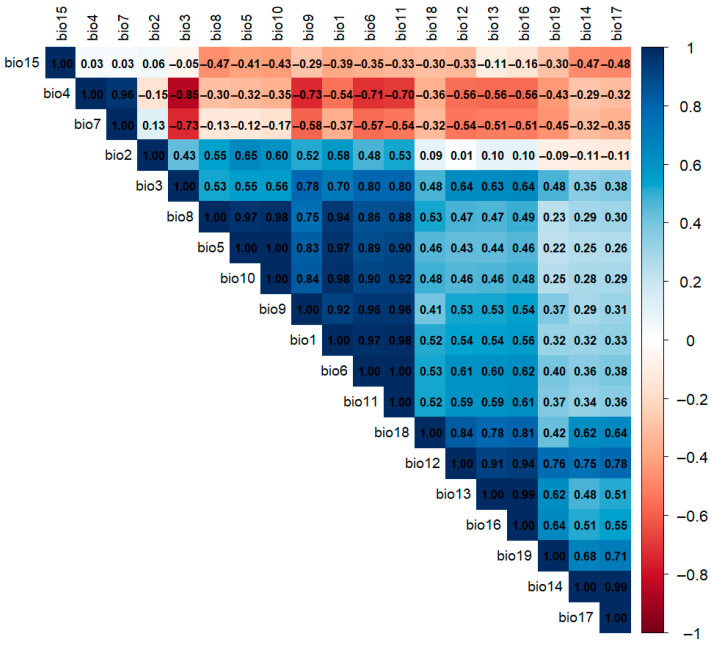
Pearson pairwise correlations for 19 climatic variables.

Nineteen climate variables were examined to select a subset on the basis of two main principles: (1) variables were excluded from the analysis if the absolute value of their correlation index was greater than 0.8 (Figure 2); and (2) the climate variables selected from (1) were divided into three groups: the temperature variable group, the precipitation variable group, and the seasonal variable group. On the basis of the climate differences between the native and invasive (China) areas of *P. canaliculata*, 1–2 important climate variables, including basic and limiting climate variables, were selected from each group. In terms of basic variables, our study considered climate variables that directly affect the survival of *P. canaliculata*, mainly temperature and precipitation; in terms of limiting variables, we mainly considered the temperature stress that may limit the northward expansion of *P. canaliculata* in the “invasion (China)” group and the drought stress that may limit the northward expansion of *P. canaliculata* in the “invasion (China)” group to the north and northwest.

The final selection of the 6 variables was as follows: annual mean temperature (Bio 1), temperature seasonality (Bio 4), minimum temperature of the coldest month (Bio 6), annual precipitation (Bio 12), precipitation of the driest month (Bio 14), and precipitation seasonality (Bio 15).

### 2.3. Climatic Niche Shift Analysis

In our study, we estimated the niche overlap based on the framework proposed by Broennimann et al. [48]. This framework has three steps: (1) calculation of the density of occurrences and of environmental factors along the environmental axes of multivariate analysis (PCA); (2) measurement of niche overlap along the gradients of this multivariate analysis; and (3) statistical tests of niche equivalency and similarity [49]. Principal component analysis (PCA) was performed with the nine selected climatic variables detailed above to represent the niche space occupied by *P. canaliculata* in the “native” and “invasive” (China) areas. Kernel density functions were applied to estimate the smoothed density of presence records and available environments along the first two axes of the PCA, preventing biases owing to the spatial resolution of the variables [26,48].

Niche overlap was calculated for “native” versus “invasive” (China) groups by means of Schoener’s *D*, which ranges from 0 to 1 [48,49]. Rödder and Engler [50] proposed the following categories: 0~0.2, no or very limited overlap; 0.2~0.4, low overlap; 0.4~0.6, moderate overlap; 0.6~0.8, high overlap; and 0.8–1.0, very high overlap.

The climatic niche shifts between the *P. canaliculata* in native and invaded ranges (China) was analyzed by the COUE scheme (a unified terminology representing niche centroid shift, overlap, unfilling, and expansion) [27]. Centroid shift measures the change in the mean niche position; niche stability measures the proportion of the nonnative niche overlapping with the native niche and reflects the tendency of a species to conserve its niche in space; niche unfilling measures the proportion of the native niche that does not overlap with the non-native niche, indicating that the species only partially fills its niche in the invaded range; and niche expansion is estimated as the proportion of the non-native niche that does not overlap with the native niche and occurs when a species colonizes new environments in the invaded ranges. Finally, we performed niche similarity and equivalence tests to evaluate the statistical significance of the observed overlap between native and Chinese ranges climatic conditions [48,49]. The niche equivalency test addresses whether two niches are equivalent by randomly reassigning occurrences from both invasive and native niches. The niche similarity test evaluates whether the invasive niche is more or less similar to the native niche than expected by chance. Both tests were based on 1000 repetitions, the native niche was fixed as a reference, and only the invasive niches were shifted. All these analyses were performed using the ecospat R package in R 4.3.1 [51].

## 3. Results

### 3.1. Predictors Responsible for Climatic Niche Shifts

**Figure 3:**
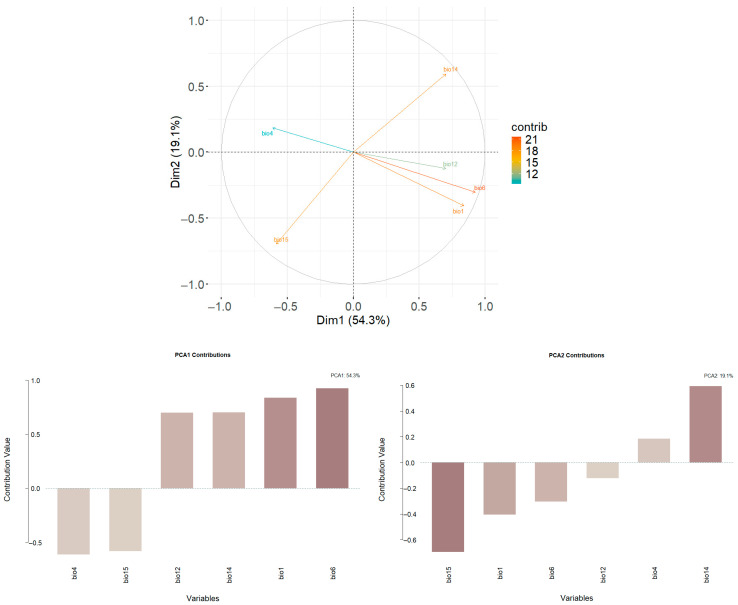
Principal component axes (PCAs) used to delimit the niche space of *P. canaliculata*. The contribution represent the loadings of the predictors on each PC axis. The first PC axis, which was mainly represented by the minimum temperature of the coldest month (Bio 6), was responsible for 54.3% of the variation. The second PC axis, which mainly reflects the precipitation seasonality (Bio 15), accounted for 19.1% of the variation. The color gradient indicates the contribution of each predictor.

PCA results demonstrated that the first two PC axes of the total niche spaces accounted for 73.4% of the variation among the six climatic predictors (Figure 3). The first PC axis, which was mainly represented by the minimum temperature of the coldest month (Bio 6), was responsible for 54.3% of the variation. The second PC axis, which mainly reflects precipitation seasonality (Bio 15), accounted for 19.1% of the variation (Figure 3). The differences in the minimum temperature of the coldest month (Bio 6) and the precipitation seasonality between the regions where the native and invasive *P. canaliculata* occur induced niche shifts.

**Figure 4:**
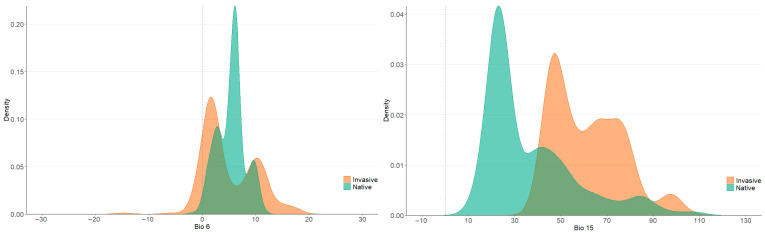
Comparisons of the climatic range between the occurrence records of *P. canaliculata* from native and invasive regions.

The Wilcoxon test revealed that, compared with native *P. canaliculata*, the invasive *P. canaliculata* was significantly more likely to occur in areas with lower minimum temperatures in the coldest month (Bio 6, *p* = 0.00037) and stronger precipitation seasonality (Bio 15, *p* = 0.0079), demonstrating significant differences (Figure 4). The results suggest that invasive *P. canaliculata* can survive in colder and more arid regions that can their native counterparts.

### 3.2. Climatic Niche Shifts

The analysis based on Schoener’s *D* and niche dynamic components (expansion, stability, and unfilling) revealed significant niche shifts in *P. canaliculata* during its invasion (Table 2). These changes were not only observed in overall patterns, but also exhibited distinct temporal dynamics.

### 3.3. Niche Overlap

The overall niche overlap between native and invasive *P. canaliculata* was remarkably low (Schoener’s *D* = 0.0467). Temporally, the niche overlap showed a pattern of initial decline followed by gradual increase; it reached its lowest value in the 2000s (Schoener’s *D* = 0.0175) before rebounding to Schoener’s *D* = 0.0418 by the 2020s. The results of similarity tests were all non-significant (*p* > 0.01), with niche similarity being lower than expected by chance, and failing to reject the null hypothesis. This finding indicates that *P. canaliculata* did not select for environmental conditions in the invaded range that were highly similar to those in its native range, demonstrating niche dissimilarity. In contrast, all niche equivalence tests yielded significant results (*p* < 0.01), rejecting the null hypothesis and confirming significant differences between the native niche and invasive niche. In summary, *P. canaliculata* exhibited a significant niche shift, rejecting the niche conservatism hypothesis for invasive species.

### 3.4. Climatic Niche Shifts Dynamics

**Figure 5:**
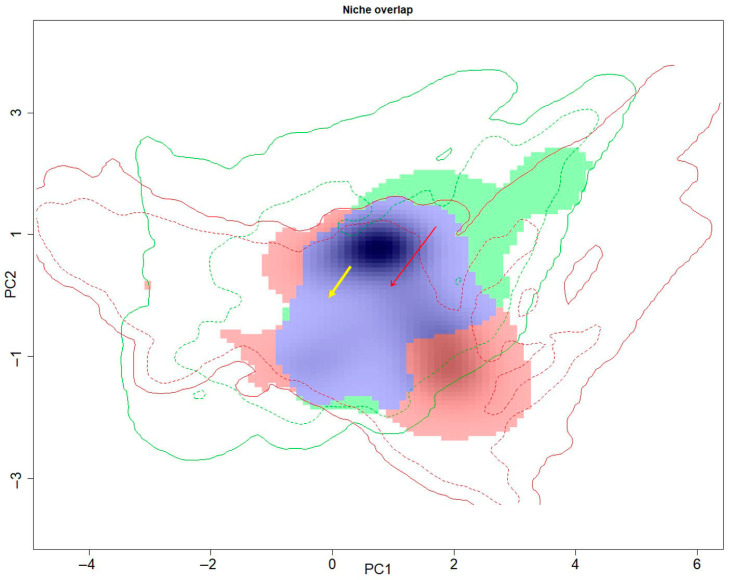
Niche dynamics of *P. canaliculata* in the “native” group versus invaded range in the “invasive” (China) group. The colored areas represent the density of occurrences, and the lines represent the available environmental background in the native (green) and invaded (red) ranges; the blue area represents the niche stability, the green area represents niche unfilling, and the red area represent niche expansion; and the arrows represent the centroid shift between the native and invaded realized niches (red solid arrow) and between the native and invaded environmental backgrounds (yellow solid arrow).

**Figure 6:**
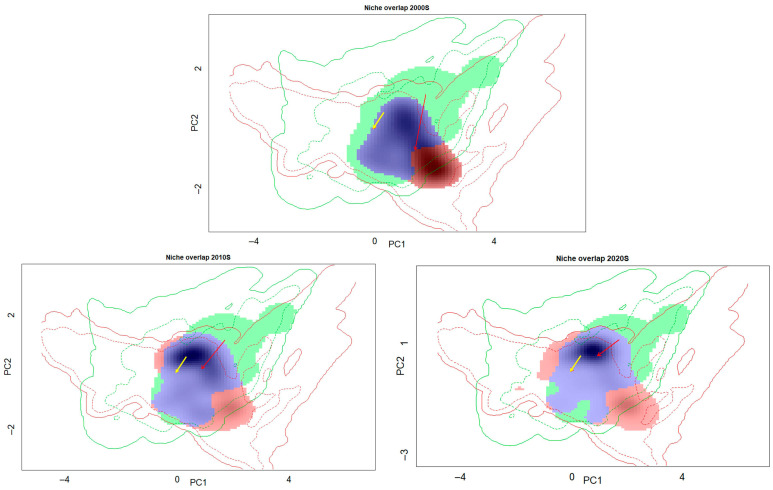
Niche dynamics of *P. canaliculata* in the “native” groups versus the invaded range in the “invasive” (China) group in different decades. Colored areas represent the density of occurrences and the lines represent the available environmental background in the native (green) and invaded (red) ranges; the blue area represents the niche stability, the green area represents the niche unfilling, and the red area represents the niche expansion; and the arrows represent the centroid shift between the native and invaded realized niches (red solid arrow) and between the native and invaded environmental backgrounds (yellow solid arrow).

The overall niche expansion, niche stability, and niche unfilling between native and invasive *P. canaliculata* were 0.1975, 0.8024, and 0.1153, respectively (Figure 5). During different time periods, the highest degree of niche expansion (0.2511) and niche unfilling (0.5315) occurred in the 2000s; the highest degree of niche stability (0.8818) occurred in the 2010s, while the degree of niche expansion and niche unfilling declined (0.1181); the lowest degree of niche unfilling (0.1916) occurred in the 2020s, while the degree of niche expansion slightly rebounded (Figure 6).

## 4. Discussion

### 4.1. The Main Variables Driving Climatic Niche Shifts in P. canaliculata

Schoener’s *D*, the niche similarity test, and the equivalence test revealed that the niche similarity between the native and invaded areas (China) of *P. canaliculata* was very limited (Schoener’s *D* was within 0.2, similarity test *p* > 0.01, equivalence test *p* < 0.01), indicating that *P. canaliculata* experienced significant niche shifts after invading China. The PCA results revealed that Bio 6 (minimum temperature of the coldest month) and Bio 15 (precipitation seasonality) were the main climatic variables driving the niche shift in *P. canaliculata*, and *P. canaliculata* significantly expanded to areas with lower temperatures and stronger precipitation seasons in the invaded area (China). This result is similar to the results obtained from the analysis of the climate adaptation of the Yunnan *P. canaliculata* population, which indicated a differentiation between the Yunnan *P. canaliculata* population and its native niche. The Yunnan *P. canaliculata* population adapted to regions with lower annual average temperatures (Bio 1) and stronger precipitation seasons (Bio 15) [21]. Some related studies conducted in other regions of Asia have obtained similar results. Matsukura et al. [15] reported that the degree of niche overlap between the Japanese *P. canaliculata* population and the native population was very limited. The lowest winter temperature (similar to that of Bio 6) was a key factor limiting the northward expansion of *P. canaliculata*. The Japanese *P. canaliculata* population adapted to low temperatures through diapause (dormancy), which is consistent with the observed expansion of cold tolerance in China. Horgan et al. [13] noted in their study of niche shifts in *P. canaliculata* in the Philippines that seasonal precipitation (similar to that in Bio 15) dominated the shift in the niche, and its niche extended to areas with more pronounced drought.

The finding that Bio 6 leads to a niche shift *P. canaliculata* indicates that its cold tolerance has increased in the invaded area (China), as *P. canaliculata* can reduce its metabolic rate and energy consumption in low-temperature environments [20]. In terms of behavioral performance, *P. canaliculata* burrows into the sediment to reduce the exposure time to cold water bodies, prevent extremely low temperatures, and increase the overwintering survival rate [15]. Another key driving variable, Bio 15, indicates that the invasive population of *P. canaliculata* in China is more adapted to environments with stronger precipitation. This is related to the increased drought tolerance of *P. canaliculata*. Studies have shown that *P. canaliculata* can survive the dry season by closing their shells or being dormant [32], whereas some invaded areas in China have distinct dry and wet seasons, which may screen individuals with greater drought tolerance. There are also studies indicating that the population of *P. canaliculata* in southern China can adapt to seasonal precipitation by reducing the metabolic rate and delaying egg laying during the dry season [19], as concentrated egg laying during the rainy season can improve the survival rate of juveniles [52]. In addition, the release of competition and natural enemies [53] may have further promoted the adaptive evolution of *P. canaliculata* in invasive areas, expanding its niche. Previous studies shown that *P. canaliculata* lacks effective competitors in Asian rice fields, leading to population outbreaks [54].

Although the invasive population of *P. canaliculata* in China is cold and drought tolerant, extremely low temperatures in winter remain the main limiting variable for their expansion to higher latitudes (such as Northeast China) [21]. In the current context of global climate change, the climate may further alter the distribution pattern of *P. canaliculata*. If the warming trend continues in the future winter (such as the increase in Bio 6), its distribution boundary may continue to shift northward. On the other hand, changes in precipitation patterns (such as an increase in extreme drought or rainstorm events) may further change suitable habitats by affecting the hydrological conditions of wetlands. The niche shifts in *P. canaliculata* indicate its strong environmental adaptability, which poses a great challenge for invasion risk assessment and management. The traditional niche model (ENM) may underestimate the potential distribution of *P. canaliculata* [26], and future research needs to combine experimental ecology (such as cold and drought tolerance testing) with genomics (such as adaptive gene screening) to reveal the molecular mechanisms of rapid *P. canaliculata* adaptation. In addition, prevention and control strategies should focus on populations with strong adaptability to low temperatures and drought, such as monitoring their overwintering survival rate in winter or strengthening agricultural drainage management in areas with strong seasonal precipitation.

In this study, we demonstrated that *P. canaliculata* exhibits adaptability to colder and drier climatic conditions within its invasive range in China. However, it is important to acknowledge that the distribution dataset compiled for this research may include records from non-natural habitats, such as experimental rice paddies and artificial reservoirs. Given that these anthropogenically modified environments could inherently favor the survival of *P. canaliculata*, their inclusion in the dataset might introduce confounding biases, despite our efforts to minimize such influences during data curation. Therefore, future investigations should not only focus on elucidating ecological mechanisms in natural habitats, but also assess population dynamics in human-altered environments to inform more comprehensive management strategies for *P. canaliculata*.

### 4.2. Climatic Niche Shift Dynamics and Invasion Strategies of P. canaliculata

In our study, we conducted a comprehensive and time-dependent analysis of the niche drift of *P. canaliculata* in its native and invaded areas (China). Overall, Schoener’s D shows that the niche overlap between the native and invaded areas (China) of *P. canaliculata* is extremely limited (Schoener’s D = 0.0467). The results of both the similarity test (*p* > 0.01) and the equivalence test (*p* < 0.01) indicate that the invasive population of *P. canaliculata* has broken free from the constraints of the native climatic variables. The high stability and significant expansion indicate that the core ecological needs of *P. canaliculata* remain conservative, but its ability to adapt to new environments is strong.

At different stages, there is significant temporal heterogeneity in the niche shifts in *P. canaliculata* between its native and invaded areas (China). Schoener’s *D* gradually increases over time, but the highest value is still far below 0.2. This phenomenon reflects the view that invasive species may remain relatively stable in core niches and exhibit greater plasticity or adaptability in marginal niches [27]. Schoener’s *D* was relatively low in the early stages, indicating that nonbiological factors filtered out individuals who were unable to adapt during the invasion of *P. canaliculata*. In the later stage, Schoener’s *D* increased, indicating that the population broke through the initial limitations through evolution or plasticity, and that the overlap with the native niche increased. In addition, human interference factors can also increase Schoener’s *D*, and agricultural activities (such as irrigation) may artificially simulate the native climate, leading to Schoener’s *D* approaching the native level. The gradual increase in Schoener’s *D* suggests that we need to further evaluate areas that were previously considered “low-risk”, such as high-latitude regions under climate change. An analysis of the expansion in the 2010s and 2020s stages revealed that Schoener’s *D* and expansion increased synchronously, indicating that the niche of *P. canaliculata* in the invaded area still expanded overall, rather than returning to its original state. In the 2000s stage, high expansion (0.2511) and high unfilling (0.5315) coexisted, which may indicate that *P. canaliculata* rapidly expanded its niche during this period, while also having a large amount of unoccupied niche space. During this stage, *P. canaliculata* may experience genetic bottlenecks and rapid adaptation [55], exploring and occupying marginal habitats through rapid diffusion and broad-spectrum environmental tolerance. This stage is often accompanied by high niche expansion and high phenotypic diversity, laying the foundation for the local adaptation of subsequent populations [56]. In the 2010s stage, stability reached its peak (0.8818), but both expansion and unfilling decreased, which may indicate that the niche of *P. canaliculata* tended to stabilize and that the expansion rate slowed during this period. During this stage, it is possible that *P. canaliculata* underwent natural selection, eliminating unsuitable genotypes and forming a new niche core. This phenomenon is consistent with the concept proposed by Colautti et al. [57] that invasive species have specific biotic and abiotic factors such as “sieving” in each invasion process, determining whether the species can enter the next stage. The results obtained from our study revealed that Bio 6, as a strong filtering variable, eliminated genotypes that were not cold-resistant, leading to a sudden increase in niche stability during the 2010s stage, whereas Bio 15 screened populations that could synchronize their reproductive cycles with the rainy season, improving population survival rates. In the 2020s phase, unfilling recharge significantly decreased, accompanied by an increase in expansion. This phenomenon indicates that *P. canaliculata* further occupied more niche space during this period, and the niche expansion slightly rebounded, which may indicate that *P. canaliculata* started to expand to a certain extent again. The invasion behavior of *P. canaliculata* shows a discontinuous and phased expansion pattern in its new environment. This model is similar to the “pulsed invasion” model proposed by Johnson et al. [58] when *Lymantria dispar* invaded North America. The concept of “pulsed invasion” emphasizes that invasion is driven by environmental fluctuations, human interference, or adaptive evolution within the population, rather than uniform linear diffusion. This invasion pattern is not common among the invasive species of freshwater gastropods, and the reason why snails exhibit this pattern may be related to special human transmission pathways (such as the spread of irrigation systems in rice-growing areas) and climate diversity in China; however, current research in this area is very rare. In addition to the aforementioned considerations, another scenario warrants attention. Arim et al. [59] posited that the diffusion dynamics of the majority of invasive species, encompassing invertebrates, demonstrate a negative feedback structure. Specifically, when the diffusion rate strays from its equilibrium state, the system employs a negative feedback mechanism to reinstate it to equilibrium. The invasion pattern of *P. canaliculata* in China, as discerned in this study, may similarly be modulated by this negative feedback structure, thereby manifesting as a non-continuous, phased invasion pattern that gravitates towards a stabilized invasion rate. Consequently, future research endeavors should incorporate a more profound and integrated analysis, taking into account the potential regulatory mechanisms that may emerge during the process of species invasion.

In addition, the invasion pattern of *P. canaliculata* conforms to the r-strategy life history characteristics, which are associated with rapid population growth, high reproductive output, and stronger environmental tolerance, and these characteristics may be associated with greater phenotypic plasticity [56]. The r-strategy life history supports the “rapid adaptor hypothesis” [60], indicating that after invading a new environment, *P. canaliculata* can rapidly undergo adaptive evolution to better adapt to selection pressures in the new environment (such as climate, soil, and biological interactions).

## 5. Conclusions

In this study, we analyzed the climatic niche shifts and their driving factors of *Pomacea canaliculata* between its native (South America) and invaded areas (China) using the COUE scheme (a unified terminology representing niche centroid shift, overlap, unfilling, and expansion), and explored their changes on a time scale. The results revealed that there has been a significant climate niche shifts between the native and invaded area (China) of *P. canaliculata*, which does not support the ”climatic niche conservatism hypothesis”. The minimum temperature of the coldest month (Bio 6) and precipitation seasonality (Bio 15) were key climatic variables driving climatic niche shift in *P. canaliculata*, indicating that the invasive population of *P. canaliculata* in China has enhanced their own cold and arid resistance. Dynamic analysis on a time scale revealed that climatic niche shift in *P. canaliculata* exhibit phased characteristics: in the 2000s, the niche rapidly expanded with a high unfilling; in the 2010s, the stability tended to stabilize and reached its peak; the unfilling significantly decreased in the 2020s, and the niche expanded again. The invasion behavior of *P. canaliculata* in China presents a discontinuous and phased expansion pattern, with strong adaptability to new environments. We also found that the climatic niche shift in *P. canaliculata* may be related to its life history strategy (r-strategy) and rapid adaptation ability, including behavioral regulation and genetic adaptation. Global climate change may further drive its expansion towards higher latitudes, while traditional niche models may underestimate its potential distribution. Future research needs to combine experimental ecology and genomics to reveal its adaptive mechanisms and provide a basis for developing prevention and control strategies. In summary, the climatic niche shift in *P. canaliculata* in China reflects its strong environmental adaptability, posing new challenges to invasion risk assessment and prevention.

## Figures and Tables

**Table 1:** 19 Climate variables.

Bio 1	annual mean temperature	Bio 11	mean temperature of the coldest quarter
Bio 2	mean diurnal temperature range	Bio 12	annual precipitation
Bio 3	isothermally	Bio 13	precipitation of the wettest month
Bio 4	temperature seasonality	Bio 14	precipitation of the driest month
Bio 5	maximum temperature of the warmest month	Bio 15	precipitation seasonality
Bio 6	minimum temperature of the coldest month	Bio 16	precipitation of the wettest quarter
Bio 7	temperature of annual range	Bio 17	precipitation of the driest quarter
Bio 8	mean temperature of the wettest quarter	Bio 18	precipitation of the warmest quarter
Bio 9	mean temperature of the driest quarter	Bio 19	precipitation of the coldest quarter
Bio 10	mean temperature of the warmest quarter		

**Table 2:** Niche overlap (Schoener’s *D*), expansion, stability, and unfilling between the “native” and “invasive” (China) ranges of *P. canaliculata*.

	Schoener’s *D*	Expansion	Stability	Unfilling
All	0.0467	0.1975	0.8024	0.1153
2000s	0.0175	0.2511	0.7488	0.5315
2010s	0.0285	0.1181	0.8818	0.3313
2020s	0.0418	0.1514	0.8485	0.1916

## Data Availability

The data presented in this study are available on request from the corresponding author since the completion of the funded project is still pending.

## Appendix A. The Data Cited from GBIF

	**Data Set**	**Link**
native distribution data of *P. canaliculata.*	iNaturalist contributors, iNaturalist (2025). iNaturalist Research-grade Observations. iNaturalist.org. Occurrence dataset https://doi.org/10.15468/ab3s5x (accessed on 2 August 2025) via GBIF.org.	https://www.gbif.org/occurrence/search?country=AR&dataset_key=50c9509d-22c7-4a22-a47d-8c48425ef4a7&taxon_key=2292582&occurrence_status=present (accessed on 2 August 2025) via GBIF.org.
	Darrigran G, Damborenea C (2024). Zoología Invertebrados—Malacología. Version 1.2. Museo de La Plata. Occurrence dataset https://doi.org/10.15468/3h7xpk (accessed on 2 August 2025) via GBIF.org.	https://www.gbif.org/occurrence/search?country=AR&dataset_key=93efdc85-10ca-448c-a3e3-01bce24479c9&taxon_key=2292582&occurrence_status=present (accessed on 2 August 2025) via GBIF.org.
	Seuffert M E, Martín P R (2024). Global records of the invasive freshwater apple snail *Pomacea canaliculata* (Lamarck, 1822). Version 1.3. Instituto de Ciencias Biológicas y Biomédicas del Sur (INBIOSUR). Occurrence dataset https://doi.org/10.15468/j4tbns (accessed on 2 August 2025) via GBIF.org.	https://www.gbif.org/occurrence/search?country=AR&dataset_key=26820b90-367e-4029-b70c-db601c591128&taxon_key=2292582&occurrence_status=present (accessed on 2 August 2025) via GBIF.org.
	Tablado A, Rodríguez D (2021). Museo Argentino de Ciencias Naturales “Bernardino Rivadavia” (MACN). Invertebrates National Collection (MACNIn). Museo Argentino de Ciencias Naturales. Occurrence dataset https://doi.org/10.15468/uuz636 (accessed on 2 August 2025) via GBIF.org.	https://www.gbif.org/occurrence/search?country=AR&dataset_key=6197c830-d9c7-11de-b793-b8a03c50a862&taxon_key=2292582&occurrence_status=present (accessed on 2 August 2025) via GBIF.org.
	Orrell T, Informatics and Data Science Center—Digital Stewardship (2025). NMNH Extant Specimen Records (USNM, US). Version 1.96. National Museum of Natural History, Smithsonian Institution. Occurrence dataset https://doi.org/10.15468/hnhrg3 (accessed on 2 August 2025) via GBIF.org.	https://www.gbif.org/occurrence/search?country=AR&dataset_key=821cc27a-e3bb-4bc5-ac34-89ada245069d&taxon_key=2292582&occurrence_status=present (accessed on 2 August 2025) via GBIF.org.
	Carnegie Museums (2025). Carnegie Museum of Natural History—Mollusks. Occurrence dataset https://doi.org/10.15468/4rfubm (accessed on 2 August 2025) via GBIF.org.	https://www.gbif.org/occurrence/search?country=AR&dataset_key=07ae2aa8-5031-4312-b26e-84a5c753daac&taxon_key=2292582&occurrence_status=present (accessed on 2 August 2025) via GBIF.org.
	Harvard University M, Morris P J (2025). Museum of Comparative Zoology, Harvard University. Version 162.476. Museum of Comparative Zoology, Harvard University. Occurrence dataset https://doi.org/10.15468/p5rupv (accessed on 2 August 2025) via GBIF.org.	https://www.gbif.org/occurrence/search?country=AR&dataset_key=4bfac3ea-8763-4f4b-a71a-76a6f5f243d3&taxon_key=2292582&occurrence_status=present (accessed on 2 August 2025) via GBIF.org.
	SysTax. SysTax—Zoological Collections. Occurrence dataset https://doi.org/10.15468/zyqkbl (accessed on 2 August 2025) via GBIF.org.	https://www.gbif.org/occurrence/search?country=AR&dataset_key=7d8ed137-1d30-42f1-8b78-12a4957e4690&taxon_key=2292582&occurrence_status=present (accessed on 2 August 2025) via GBIF.org.
	Denver Museum of Nature & Science Marine Invertebrate Collection https://doi.org/10.15468/lyw1fq (accessed on 2 August 2025) via GBIF.org.	https://www.gbif.org/occurrence/search?country=AR&dataset_key=5a6977c1-ece6-44b8-b71d-43cb1d4e0919&taxon_key=2292582&occurrence_status=present (accessed on 2 August 2025) via GBIF.org.
	The International Barcode of Life Consortium (2024). International Barcode of Life project (iBOL). Occurrence dataset https://doi.org/10.15468/inygc6 (accessed on 2 August 2025) via GBIF.org.	https://www.gbif.org/occurrence/search?country=AR&dataset_key=040c5662-da76-4782-a48e-cdea1892d14c&taxon_key=2292582&occurrence_status=present (accessed on 2 August 2025) via GBIF.org.
invasive distribution (in China) data of *P. canaliculata*	European Bioinformatics Institute (EMBL-EBI), GBIF Helpdesk (2025). INSDC Sequences. Version 1.148. European Nucleotide Archive (EMBL-EBI). Occurrence dataset https://doi.org/10.15468/sbmztx (accessed on 2 August 2025) via GBIF.org.	https://www.gbif.org/occurrence/search?country=CN&dataset_key=d8cd16ba-bb74-4420-821e-083f2bac17c2&taxon_key=2292582&occurrence_status=present (accessed on 2 August 2025) via GBIF.org.
	iNaturalist contributors, iNaturalist (2025). iNaturalist Research-grade Observations. iNaturalist.org. Occurrence dataset https://doi.org/10.15468/ab3s5x (accessed on 2 August 2025) via GBIF.org.	https://www.gbif.org/occurrence/search?country=CN&dataset_key=50c9509d-22c7-4a22-a47d-8c48425ef4a7&taxon_key=2292582&occurrence_status=present (accessed on 2 August 2025) via GBIF.org.
	Seuffert M E, Martín P R (2024). Global records of the invasive freshwater apple snail *Pomacea canaliculata* (Lamarck, 1822). Version 1.3. Instituto de Ciencias Biológicas y Biomédicas del Sur (INBIOSUR). Occurrence dataset https://doi.org/10.15468/j4tbns (accessed on 2 August 2025) via GBIF.org.	https://www.gbif.org/occurrence/search?country=CN&dataset_key=26820b90-367e-4029-b70c-db601c591128&taxon_key=2292582&occurrence_status=present (accessed on 2 August 2025) via GBIF.org.
	The International Barcode of Life Consortium (2024). International Barcode of Life project (iBOL). Occurrence dataset https://doi.org/10.15468/inygc6 (accessed on 2 August 2025) via GBIF.org.	https://www.gbif.org/occurrence/search?country=CN&dataset_key=040c5662-da76-4782-a48e-cdea1892d14c&taxon_key=2292582&occurrence_status=present (accessed on 2 August 2025) via GBIF.org.
	Zhou J, Wong L (2025). CBCGDF CCAfa Volunteer Observation Archive. Version 1.198. China Biodiversity Conservation and Green Development Foundation. Occurrence dataset https://doi.org/10.15468/wxze8b (accessed on 2 August 2025) via GBIF.org.	https://www.gbif.org/occurrence/search?country=CN&dataset_key=1b26c417-366a-4eb0-a212-6ee8035d83fb&taxon_key=2292582&occurrence_status=present (accessed on 2 August 2025) via GBIF.org.
	Wong L, Chen S, Chen S (2025). CBCGDF-Green-Youth. Version 1.42. China Biodiversity Conservation and Green Development Foundation. Occurrence dataset https://doi.org/10.15468/8dteut (accessed on 2 August 2025) via GBIF.org.	https://www.gbif.org/occurrence/search?country=CN&dataset_key=98d8d571-f83f-4076-8a41-86056198a969&taxon_key=2292582&occurrence_status=present (accessed on 2 August 2025) via GBIF.org.
	naturgucker.de. NABU| naturgucker. Occurrence dataset https://doi.org/10.15468/uc1apo (accessed on 2 August 2025) via GBIF.org.	https://www.gbif.org/occurrence/search?country=CN&dataset_key=6ac3f774-d9fb-4796-b3e9-92bf6c81c084&taxon_key=2292582&occurrence_status=present (accessed on 2 August 2025) via GBIF.org.
	Illinois Natural History Survey (2025). Illinois Natural History Survey- Mollusk Collection. Occurrence dataset https://doi.org/10.15468/cu4scd (accessed on 2 August 2025) via GBIF.org.	https://www.gbif.org/occurrence/search?country=CN&dataset_key=763f9aee-060c-4d71-a538-16c383e53a4d&taxon_key=2292582&occurrence_status=present (accessed on 2 August 2025) via GBIF.org.
